# Facial Neuralgia

**Published:** 1862-03

**Authors:** J. S. Latimer


### Selections.

FACIAL NEURALGIA. *By J. S. Latimer, D. D. S.*—Neuralgia, from *neuros*, a nerve, and *algos*, pain, means, strictly, pain in a nerve; and, though all pain is felt in the nerves or *through* the nerves, yet the nomenclature of our fathers is pretty well understood, and we will not quarrel with it. We have, also, the name tic doloreux, or painful tic. The name probably came from the fact that a convulsive twitching is sometimes seen in the faces of persons affected with this painful disease.

Concerning the causes of Neuralgia, many and opposing theories have been entertained, and, of course, the treatment based on those theories has varied as much.

The necessity of understanding the true cause in any given instance is fully shown in the fact that some, at one time, believed the facial nerve the seat of the affection, and resorted to excision of its trunk at the stylo-mastoid foramen, by which the muscles of one side of the face were paralyzed, while the pain continued unabated.

Neuralgia is divided by some into pure and intermittent. The former "is not dependent on any other affection," while the latter is consequent on malarious poisoning, and is often the only symptom of intermittent fever, as it is called. But I doubt not we often find the disease symptomatic from indigestion, uterine affections and abnormal conditions of various organs not at the time under the influence of miasma.

Dr. Carnochan, in an able paper on the subject of Facial Neuralgia, takes the ground that the disease (pure neuralgia, of course,) is generally located in the second branch of the fifth pair, called the superior maxillary nerve, and that it is anterior to the foramen rotundum from which the nerve makes its exit after leaving the Casserian ganglion.

Among the causes of injury to the nerve, he enumerates, "1, prolonged irritation upon the periphery; 2, exposure; 3, injuries; 4, tumors; 5, diseases of the teeth; 6, pressure resulting from the periosteal or osteal thickening of the osseous foramina or canals; 7, sudden suppression of any of the important secretions, as of the catamenial discharge."

Here let us briefly examine the anatomy of the Trigeminus nerve. You will recall that it arises by two roots, the anterior or motor, and the posterior or sensory; the latter being much the larger, and having a ganglion upon it. But if you will excuse the comparison, I will liken this nerve to the telegraph system. The sensory line terminates in the office of the commander-in-chief, anatomically called the medulla oblongata. This wire is composed of a hundred or more small wires, each insulated from the others, as in the Atlantic cable. It emerges from the pons varolii where the pons joins the crus cerebelli, passes forward and enlarges into the ganglion of Casser, which rests in a depression of the upper surface of the petrous portion of the temporal bone.

This ganglion is important as the beginning of the grand trunk line, in which three important converging lines meet, and through which all communications are forwarded to the sensorium.

The first of the three lines meeting in the ganglion, is the ophthalmic which physiologically arises in several branching lines from the upper eyelid, integuments of the forehead, conjunctiva, lachrymal gland, the mucous membrane, and the muscles of the nose, the ball of the eye, etc. From these minor stations lines converge to form the ophthalmic branch, which enters the cavity of the skull through the sphenoidal fissure.

The third line is formed by the convergence of several small lines, commencing in the inferior teeth, the mucous membrane of the mouth, the tongue, tonsils, pharynx, gums, integument of the lips and chin, the parotid gland, the integument of the temple, etc.

But most important, perhaps, for our present consideration is the second branch or superior maxillary nerve. This line has ramifying branches and terminal offices in all the upper teeth, the eyelids, nose, lips, cheeks, the lining membrane of the antrum, gums, mucous membrane of the nares and palate, the integument of the temple and side of the face.

From all these points branches converge to form the orbital, the spheno palatine, the posterior, anterior and middle dental, and the infra-orbital lines, which unite anterior to the foramen rotundum, form the second branch and pass through to the ganglion.

All these lines are under the control of one company, and, as has been intimated in the description of the sensory root, each terminal office has a wire running entirely through to the sensorium. Other lines, as the portio dura and the great sympathetic, have long occupied several offices in common with the trigeminal, with mutual advantage to all parties concerned.

As yet, we have barely mentioned the fact that the tri-facial has a motor root. This root is very small, arises from the pyramidal body of the medulla oblongata, passes close to and beneath the sensory root and ganglion without uniting with them, and emerges from the chamber through the foramen ovale, where it unites with the inferior maxillary branch just beyond the otic ganglion. Hence, of the fifth pair, only the inferior maxillary branch has control over muscular motion, the others being merely nerves of special sense and sensibility.

Now, when we consider the intimate relation existing between the great sympathetic and the fifth pair, we are the better able to comprehend how a dispatch to the ganglion of Meckel (which office is also occupied by an agent of the tri-facial line), giving account of intestine difficulties in the hypogastric region, would be likely to produce considerable commotion all along the line of the second branch.

I am not aware that any one has satisfactorily explained the precise manner in which sympathy with a distant organ produces disease in the body of a nerve. We must, at present, be content to know that such is the case.

There is little doubt in my mind that miasma affects primarily the functions of the liver, or other important viscera, and that pain in distant parts is entirely symptomatic, though it is no doubt true that where the irritation of a nerve is thus continued for a considerable time, the ultimate is lesion of its tissue.

It behooves us, then, when a case presents with pain in the region traversed by the tri-facial, to examine well the symptoms. If the pain is sharp and darting, following the track of the nerve, appearing and disappearing suddenly, we may be sure we have a case of neuralgia. Our next object is to discover and remove the cause.

As the teeth are often responsible for the mischief, we first examine them, and if an exposed pulp or ulcerated fangs are present, they should at least be regarded with suspicion. If the pain is of the regular intermittent type, we suspect it to be symptomatic, in part, at least. If, after examination, no sign of disease in the teeth, gums, alveoli or antra is discovered, we may safely infer that the primary cause is not and has not been confined to the mouth ; though, as Dr. Carnochan declares, the cause *may* be exostosis of the osseous canals or foramtna, or, quite as possible, thickening of the osteal or periosteal membrane along the nerve track. But these latter causes are rather obscure, and can not always be discovered.

It is no doubt true that the effusion of lymph, which often terminates in inflammation, may, by endosmotic action, penetrate the neurilemma and disease the nerve tissue; indeed, the vascular, engorged and thickened condition of portions of the superior maxillary nerve removed by Dr. Carnochan, seems to incontrovertibly prove this fact. It is more than likely, too, that disease of the nerve tissue often remains long after the removal of the inflammatory cause. Hence the importance of careful inquiry of the patient concerning blows, falls, and other exciting causes, though of a comparatively remote period.

By some, odontitis or true toothache is pronounced neuralgia. This, it seems to me, is objectionable, because the disease does not extend to any portion of the nerve beyond the immediate neighborhood of the inflamed pulp, and because the pain is not of the neuralgic character. Of course, the periphery of some nerve is injured whenever inflammation of any part of the body exists; yet we do not necessarily have neuralgia. That exists from exposure of the dental pulp only when prolonged irritation of the peripheric termination, aided by favoring circumstances, induces disease of the body of the nerve. If the causes are slight, or the constitution rather unfavorable to the disease, or if those causes have existed but a brief period, the disease may subside on the removal of the primary cause.

That two or more causes may exist at the same time is well known. In the New York "Dental Recorder" for March, 1847, a case is reported, in which facial neuralgia of a regular intermittent type was cured by the removal of several ulcerated fangs, and that, too, after constitutional treatment had been tried in vain. It is *possible* that *all* the causes of facial neuralgia enumerated by Dr. Carnochan might be found in one case, and we are not to rest content with a slight examination.

In illustration of the necessity of *thorough* search for the cause of disease, let me give you a case from my own practice.

Mr. T., student, attending his second course of lectures at a medical school of this city, came to me last week, suffering from what he called facial neuralgia. Mr. T. is of the nervo-lymphatic temperament, aged about twenty-five, and healthy. The pain was not darting, but presented more the character of odontitis, extending along the line of the superior dental nerves, and forward to the median line, though seeming to center in the second superior bicuspid of the right side. The anterior teeth were sound, as were the bicuspids, though the second bicuspid was tender under percussion, and showed evidence of periosteal irritation. The anterior approximal surface of the first large molar had a large decay, but a fine probe failed to detect an exposure, though tr. iodine applied to the cavity increased the pain considerably. It, too, was sensitive under percussion, though not in the same degree. The second multicuspid was sound and every way healthy, but the dens sapientia was chalky, stood without the line and pointing toward the cheek, while on the grinding surface was an irregular and deep cavity, but in which I was unable to detect an exposure. The other teeth had no exposed pulps.

Under the circumstances, I ordered a cathartic, a leech to the second bicuspid, and applied the arsenical paste to the first multicuspid. The next morning the patient called, saying the pain was increased, and that he had not been able to sleep until he "obtained liquor and got drunk." The tooth to which paste was applied remained as before, but the difficulty with the second bicuspid was increased, the leeching having failed to produce the least mitigation of suffering. Excavating the cavity of the large molar considerably further, I became satisfied that there had been no exposure of the pulp, and that the paste had not yet acted on it. The teeth were again thoroughly examined, but without result. At this juncture the patient impatiently demanded the removal of the suspected bicuspid, and I, baffled in my effort to find the cause, weakly, yea, wickedly consented, and extracted it. Both the pulp and periosteum were slightly congested, but, with the exception of two epienamel decays on the approximating surfaces, it was sound. The pain continued, and would not yield to laudanum in the alveolus.

We then concluded that though the wisdom-tooth did not *seem* to be the cause of the trouble, it must soon become painful, and its removal *might* give relief; and so, as the physician, who has exhausted the pharmacopœia in vain, comes at last to calomel as the *dernier resort*, we removed the dens sapientia, when lo, the pain was all gone and the tenderness of the other teeth on that side immediately disappeared! On the buccal surface of the culprit, and which had been entirely covered by a loose and slightly-turgid margin of the gum, was a large decay in which the pulp was exposed. One important symptom which I omitted to mention in the proper place, was the pain caused in giving any lateral motion to the lower jaw.

It is by such severe lessons as this, gentlemen, that we are humbled and prevented from turning our ears from instruction by the conceit which unbroken success is likely to cultivate, unless we impute that success to the overruling providence of the Omnipotent.

But you will pardon the digression, made for the purpose of showing the necessity of following Davy Crockett's maxim, "*Be* SURE *you are right, then go ahead.*"

Another case may serve to show the difficulty of discovering the cause of tic doloreux. Some ten days since, an Irish laborer came to me for the removal of the first superior right multicuspid. Temperament, bilio-lymphatic; age, about thirty-five. Had been unable to work for three weeks from pain in the right side of the upper jaw and head. Pulse strong and regular; tongue firred slightly; the breath smelling strongly of cheap whisky. The jaws were large and the teeth all sound, with the exception of a slight cavity on the buccal surface of the first superior multicuspid. No periosteal tenderness, but much tartar and some turgescence of the gums in consequence. He had already employed three physicians, who evidently suspected the existence of hydrocephalus.

The disease had been developed by a cold, from which the patient had suffered, and was evidently neuralgia of the second branch of the fifth pair; but, being unable to determine the primary cause, I filled the cavity before mentioned, with Bevin's filling, and sent the patient to Dr. Carnochan, with the result of my examination, since which I have not heard from the case.

As yet, I have not been able to learn any method of distinguishing immediately the disease with a central origin, (that is, when the trunk of the nerve has become permanently diseased and changed in structure,) from that with a peripheric or reflex origin. On this point I want light.

Mr. Lobb, in a paper read before the Harveian Society of London, ascribes idiopathic peripheral neuralgia to atrophy, by which the nerve is prevented from conducting normal sensations to the brain, and by which the polarity of the nerve is affected, thus giving the idea of pain in the diseased portion. Mr. Lobb then proceeds to argue in favor of the continuous galvanic current as the only method of restoring the lost polarity and of stimulating the nerve to healthy nutrition. Dr. Carnochan, however, believes that cure can be effected only by the removal of the ganglion of Meckel, or its insulation from the encephalon. He describes three or four formidable operations, in which he exsected the trunk of the second branch as far back as the foramen rotundum. All these cases were at least temporarily successful; though Dr. Jas. R. Wood, at a meeting of the New York Pathological Society, held about the beginning of 1860, says that in one of the cases reported by Dr. Carnochan, and in which he removed the ganglion of Meckel, the pain subsequently returned. An inch or more of the trunk has been exsected and afterward the trunk has reunited and sensibility has been restored. Excision was performed by Dr. Mott in one case thirty-six times, with temporary relief in each instance.

When the operation of exsection is to be performed on the inferior maxillary branch, the soft parts are dissected off so as to expose the external plate of the jaw near the angle, and anterior to the posterior dental foramen. With a small trephine, enough of the external plate is removed to expose the nerve, which is then easily exsected. The hemorrhage is generally subdued without resorting to the actual cautery.

Dr. E. Andrews, in a lecture published in the *Chicago Medical Examiner*, after endorsing Dr. Carnochan's theory of pressure upon the trunk of the nerve in the foramina, says that other tissues can accommodate themselves to continuous pressure, but nerve trunks under such circumstances become permanently troublesome. "The treatment," he remarks, "should be in the direction to remove the constitutional diathesis on which the local constriction depends; as for instance, the rheumatic, syphilitic, or malarious influences. If this does not suffice and the pain continues, you may resort to surgical interference."

Dr. A. Wood says, that in Edinburgh the use of narcotics injected under the skin in the vicinity of the pain is almost universal. Mr. Brenchley says that the Germans consider muriate of ammonia very valuable in facial neuralgia. It is given in doses of half a drachm every hour,in camphor mixture.

M. Vautier reports a case in which facial neuralgia, accompanied by deafness, was cured by the extraction of a wisdom tooth.

Dr. Van Archen, of Bogota, New Granada, reports an interesting fact relative to facial neuralgia caused by tobacco. He says, "I have to mention a peculiar kind of neuralgia occurring only in people who have worked for years in tobacco factories, besides being habitual smokers. In these, the body becomes so thoroughly saturated with nicotin, that occasional twitching of the muscles of the face occurs, which ultimately becomes an agonizing pain." In these cases, antispasmodics and narcotics have no curative effect, but they generally yield to the following formula:

℞ Sulphatis quinine grs. viij.
Pulveris cinehonæ,
Terri carbonatis a a ℈j.

M. Divide in four powders. S. One every six hours.

Our specialty confines our operations to the mouth, with a few exceptions, and our business is to decide whether the teeth and their surroundings are responsible for the disease, thus assisting the general surgeon into whose hands the case properly falls.

It is proper for me to state, gentlemen, in closing this paper, that want of time has prevented me from making it shorter by ridding it of much matter that may be irrelevant, and correcting the verbiage.—*Vulcanite.*

